# Association of Mu opioid receptor (A118G) and BDNF (G196A) polymorphisms with rehabilitation-induced cortical inhibition and analgesic response in chronic osteoarthritis pain

**DOI:** 10.1016/j.ijchp.2022.100330

**Published:** 2022-09-20

**Authors:** Fernanda de Toledo Gonçalves, Kevin Pacheco-Barrios, Ingrid Rebello-Sanchez, Luis Castelo-Branco, Paulo S. de Melo, Joao Parente, Alejandra Cardenas-Rojas, Isabela Firigato, Anne Victorio Pessotto, Marta Imamura, Marcel Simis, Linamara Battistella, Felipe Fregni

**Affiliations:** a Departamento de Medicina Legal, Ética Médica e Medicina Social e do Trabalho, Laboratório de Imunohematologia e Hematologia Forense (LIM40), Hospital das Clínicas da Faculdade de Medicina da Universidade de São Paulo (HC da FMUSP), São Paulo, Brazil; b Neuromodulation Center and Center for Clinical Research Learning, Spaulding Rehabilitation Hospital and Massachusetts General Hospital, Harvard Medical School, Boston, USA; c Universidad San Ignacio de Loyola, Vicerrectorado de Investigación, Unidad de Investigación para la Generación y Síntesis de Evidencias en Salud, Lima, Peru; d Instituto de Medicina Física e Reabilitação, Hospital das Clinicas HCFMUSP, Universidade de São Paulo, São Paulo, Brazil; e Faculdade de Medicina, Universidade de São Paulo, São Paulo, Brazil

**Keywords:** Chronic pain, Osteoarthritis, Polymorphism, Cortical excitability

## Abstract

**Background/objective:**

Chronic pain due to osteoarthritis (OA) is a prevalent cause of global disability. New biomarkers are needed to improve treatment allocation, and genetic polymorphisms are promising candidates.

**Method:**

We aimed to assess the association of OPRM1 (A118G and C17T) and brain-derived neurotrophic factor (BDNF [G196A]) polymorphisms with pain-related outcomes and motor cortex excitability metrics (measured by transcranial magnetic stimulation) in 113 knee OA patients with chronic pain. We performed adjusted multivariate regression analyses to compare carriers versus non-carriers in terms of clinical and neurophysiological characteristics at baseline, and treatment response (pain reduction and increased cortical inhibitory tonus) after rehabilitation.

**Results:**

Compared to non-carriers, participants with polymorphisms on both OPRM1 (A118G) and BDNF (G196A) genes were less likely to improve pain after rehabilitation (85 and 72% fewer odds of improvement, respectively). Likewise, both carriers of OPRM1 polymorphisms (A118G and C17T) were also less likely to improve cortical inhibition (short intracortical inhibition [SICI], and intracortical facilitation [ICF], respectively). While pain and cortical inhibition improvement did not correlate in the total sample, the presence of OPRM1 (A118G) and BDNF (G196A) polymorphisms moderated this relationship.

**Conclusions:**

These results underscore the promising role of combining genetic and neurophysiological markers to endotype the treatment response in this population.

## Introduction

Knee osteoarthritis (OA) is a prevalent cause of worldwide disability and decreased quality of life mainly due to chronic pain (Blyth et al., 2019; Courtney et al., 2012). Studies have shown that pain chronicity is associated to maladaptive neuroplastic changes in brain networks commonly associated with central sensitization (Guler et al., 2020; Willett et al., 2020).

One of the neuroplastic alterations present in chronic pain specifically in knee OA are the changes in cortical excitability showed by transcranial magnetic stimulation (TMS) markers (resting motor threshold [rMT], motor evoked potential [MEP], cortical silent period [CSP], short intracortical inhibition [SICI], and intracortical facilitation [ICF]) (Caumo et al., 2016; Kittelson et al., 2014; Simis, et al., 2021). Lower cortical inhibition was associated with less knee degeneration severity, lower age, and higher pain intensity in patients with chronic knee OA (Simis, et al., 2021). Previous research have shown that reduced intracortical inhibition is present in patients with motor disability and in other chronic pain conditions compared to healthy subjects (Candido Santos et al., 2020; Demirtas-Tatlidede et al., 2015; Hollins et al., 2020; Miller et al., 2014). Thus, TMS metrics are promising pain biomarkers for chronic pain phenotypification and prediction of treatment response.

Genetic polymorphisms may also be potential pain biomarkers. Besides the limited evidence for their clinical relevance, the opioid Receptor Mu 1 (*OPRM1)* gene (A118G [rs1799971] and C17T [rs1799972]) and brain-derived neurotrophic factor (*BDNF* gene – G196A [rs6265]) polymorphisms have been identified in musculoskeletal chronic pain (Zorina-Lichtenwalter et al., 2016), and may be used to understand the high variability and individual treatment response in chronic pain populations. The genetic mutation of the *OPRM1* gene has been associated with less expression of Mu-opioid receptors (MOR) (Peciña et al., 2015); therefore, carriers could have an altered pain perception, analgesia response, response to stressors, and risk for opioid addiction (Mague & Blendy, 2010). Moreover, BDNF might be considered a pain modulator since it protects the neurons from adversity, such as painful stimulus, and participates in the synaptic plasticity of pain modulation circuits (Caumo et al., 2016; Generaal et al., 2016; Haas et al., 2010; Mague & Blendy, 2010). The mutation of the *BDNF* gene reduces its secretion, thus decreasing its effects on the nervous system, likely including potential pain and analgesic modulatory processes (Egan et al., 2003).

However, for the best of our knowledge, the influence of *OPRM1* and *BDNF* polymorphisms in cortical excitability in chronic pain, and its relationship with pain-related outcomes and analgesic response were not yet explored and their clinical application's evidence is very limited. Most of the studies on the field included only clinical assessments and have a small sample size (Cash et al., 2021; Di Lazzaro et al., 2015; Lee et al., 2013).

Therefore, we aim to assess the association of *OPRM1* (A118G and C17T) and *BDNF* (G196A) polymorphisms with pain-related outcomes and cortical excitability metrics in chronic knee OA. Besides, we test whether carriers have differential responses to rehabilitation treatment compared to non-carriers, and whether the polymorphisms moderate the association between pain response and cortical excitability changes. We hypothesized carriers will have less cortical inhibition and smaller analgesic response.

## Methods

This is a cross-sectional analysis from the DEFINE study,  a prospective cohort following patients with chronic knee OA. The study protocol was approved by HC FMUSP Ethics Committee for Research Protocol Analysis (CAAE: 86832518.7.0000.0068) and is available with detailed information (Simis, et al., 2021).

### Study Procedures

We included 113 subjects from the rehabilitation program of the Instituto de Medicina Física e Reabilitação (IMREA) at Hospital das Clínicas da Faculdade de Medicina da Universidade de São Paulo (HC FMUSP). Those interested were screened and only participated after signing the consent form. Subjects had to meet the eligibility of IMREA's rehabilitation program to be enrolled in the study. The inclusion criteria also involved having (i) ≥ 18 years old; (ii) clinical and radiological confirmation of diagnosis; (iii) clinical stability. Individuals with active OA and clinical manifestations in other joints besides the knee were not included.

### IMREA rehabilitation program

The IMREA rehabilitation program is a multidisciplinary pragmatic outpatient program for adults with OA. The program is located within a tertiary care center. An individualized approach characterizes this program, considering the symptoms severity and the patients’ functional status. Specifically we used the following rehabilitation methods: Fischer paraspinous block, neuromuscular electrical stimulation (NMES) of the vastus medialis muscle, focal shock wave therapy (SWT) and radial SWT over painful areas. It is important to note that all patients involved in the research received the same type of treatment as patients who are not participating in the research, therefore, this program reflects the treatment variability of a real-world rehabilitation program.

### Assessments

Clinical and neurophysiological assessments for all subjects were performed before starting the IMREA program and immediately at the end of it (see protocol).

#### Clinical assessments

Questionnaires were applied by a trained physician. We collected demographic and OA disease information. In addition, validated clinical scales were used such as visual analogue scale for pain intensity, WOMAC pain subscale, anxiety, depression, catastrophizing, and a short cognitive scale. The description of all the questionnaires is available on the published protocol (Simis, et al., 2021).

#### Neurophysiological assessment

We performed a transcranial magnetic stimulation (TMS) assessment over the motor cortices. We used the Magstim Rapid® stimulator (The Magstim Company Limited, UK) with a 70-mm coil in a figure-of-eight (at 45 degrees of the scalp) to perform all TMS assessments. The investigator assessed the coil direction without neuronavigation. The electromyography (EMG) assessment was performed bilaterally with Ag/AgCl electrodes positioned in the first dorsal interosseous muscles of both hands and the grounding electrode on the wrist (Malcolm et al., 2006).

A bilateral upper limb assessment was performed using anatomical references for motor cortex localization as specified in our protocol. We determined the hotspot as the location with the highest and most stable motor evoked potential (MEP) amplitudes over the FDI. The resting motor threshold (rMT) was the minimum intensity for a single TMS pulse on the hotspot to generate an MEP, with at least 50μV peak to peak amplitude, in 50% of attempts (Rossini et al., 2015). We performed the following measures: MEP (intensity at 120% of rMT, we calculated the peak-to-peak amplitude), cortical silent period (CSP), which represents the temporary suppression of electromyographic activity during a sustained voluntary contraction. Furthermore, we performed paired-pulse protocols of intracortical inhibition (SICI), assessed by interstimulus intervals of 2 ms; and intracortical facilitation (ICF) assessed by 10 ms interim stimulus intervals (Rossini et al., 2015). Ten randomized stimuli were applied at each interval and the average were calculated.

For the TMS neurophysiological measurements, we calculated the rMT, CSP, SICI, ICF, and MEP. The SICI is reported as % of inhibition $=1-\frac{\text{MEP}\;2\text{ms}\;\;x\;100}{\text{MEP}\;\text{baseline}}$; thus, higher values of SICI indicates higher intracortical inhibition.

Then, we calculated a bi-hemispheric average of those metrics. This approach can be justified due to the bi-hemispheric nature of pain perception(Schwenkreis et al., 2003); besides, most of the sample includes patients with bilateral knee OA. TMS data were recorded and stored in a computer for offline analysis.

#### Genotyping

Blood samples (5 mL) were collected in EDTA (Ethylenediaminetetraacetic acid) and DNA (deoxyribonucleic acid) was isolated by the salting-out process, according to (Miller et al., 1988) and stored at −80 °C before the genotyping. Next, DNA samples were qualified and quantified using a NanoDrop™ 2000 spectrophotometer (Thermo Fisher Scientific, Waltham, MA, USA). An A260/A280 ratio between 1.8 and 2.2 was used to classify the samples as high genomic DNA quality. Genotyping of *OPRM1* (A118G/rs1799971 and C17T/ rs1799972) and *BDNF* (G196A/rs6265) polymorphisms was determined by TaqMan® SNP Genotyping Assays (Applied Biosystems, Foster City, CA, USA). The primers and probes were predesigned assays by Applied Biosystems, and genotyping was performed on the StepOnePlus™ instrumentation platform (Applied Biosystems, Foster City, CA, USA), according to the manufacturer's recommendations. Positive and negative controls were used in each genotyping assay plate, and the results of the 10% of the samples randomly selected (including positive controls) were confirmed by genome sequencing (Sanger et al., 1977) in ABI 3130 Genetic Analyzer Applied Biosystems®. Carrier status was defined as individuals carrying one or two copies of the variant allele; and non-carriers as individuals homozygous for the variant allele.

### Statistical analysis

For baseline descriptive statistics, we used frequency and percentage to summarize categorical variables, and mean and standard deviation for continuous data. After assessing normality with graphical and statistical tools (Shapiro-Wilk Test), we used the appropriate statistical for baseline comparisons (polymorphisms carriers vs non-carriers) using t-test for continuous variables and chi-square test for categorical variables. Additionally, we ran adjusted baseline comparisons (for age, sex, and OA radiographic severity), using multivariate regression models.

Moreover, we assessed the association between polymorphism status (carriers vs non-carriers) with pain (VAS) and TMS metrics changes after rehabilitation (post-treatment values minus baseline values). We defined pain response as a change of 2 or more points in VAS after treatment (=1) and non-response as less than 2 points (=0) since several studies reported 2 points in VAS as clinically important difference (Dworkin et al., 2008). Similarly, we categorized changes in TMS metrics using the median from the variable distribution as cut-off and the direction towards cortical inhibitory tonus as categorization rule. For SICI and CSP, higher values after treatment (e.g., higher cortical inhibition) was considered an improvement (=1). For rMT, MEP, and ICF, lower values after treatment (e.g., less cortical excitability/facilitation) was considered an improvement (=1). Then, we carried out univariate and multivariate logistic regressions. The dependent variables were pain or TMS metrics improvements, and the main independent variable was the polymorphism status (carriers=1, non-carriers=0). To avoid overfitting and collinearity, we tested each polymorphism in a separate model. Also, we adjusted the models for age, sex, treatment duration, OA severity, and depression, based on previous results that suggested important risk of confounding (Simis, Imamura, de Melo, Marduy, Pacheco-Barrios, et al., 2021). To test the models’ goodness-of-fit we performed the Hosmer-Lemeshow test. We reported the odds ratios and their 95% confidence intervals for the odds of improvement by polymorphism status for both unadjusted and adjusted models.

Finally, we tested whether the polymorphism status is a moderator in the relationship between pain and cortical inhibition improvement. We implemented multivariate linear regression models with inhibitory TMS metrics continuous changes (SICI and CSP) as dependent variable and continuous pain changes and the interaction of pain improvement*polymorphism status as independent variables. We considered an alpha level of 0.05 as threshold for statistically significance. The analyses were performed in STATA 17.

## Results

### Sample characteristics

Data for this article were collected from a cohort of 113 patients with chronic knee OA. The sample was composed mostly of females (83%), with a mean age of 68.6 ±9.5 (Table 1), an average VAS pain of 5.5 ±2, and mainly bilateral symptoms. Of those, we analyzed 101 DNA samples viable for *OPRM1* genotyping assays, 12 subjects were excluded due to sample quality. For the A118G polymorphism, 79 were non-carriers (AA), 19 were heterozygous G carriers (AG), and 3 were homozygous G carriers (GG). For the C17T polymorphism, 82 were non-carriers (CC), 18 were heterozygous T carriers (CT), and only 1 was a homozygous T carrier (TT) (Table 2). To analyze the G196A polymorphism on the *BDNF* gene we had 99 viable genotyping samples, of which 72 were non-carriers (GG), 26 were heterozygous A carriers (GA), and 1 homozygous A carrier (AA) (Table 2). The prevalence were 21.8%, 18.8%, and 27.3% for A118G, C17T, and G196A polymorphisms, respectively.

**Table 1:** Baseline sociodemographic characteristics of knee OA study participants.

**Demographics**	**All Knee OA subjects (N = 113)**
Age	68.65 ± 9.45
Gender (%)	
Male	19 (16.81%)
Female	94 (83.19%)
Ethnicity	
White	72 (63.72%)
Black	13 (11.5%)
Brown	22 (19.47%)
Asian	6 (5.31%)
BMI	31.99 ± 5.3
Education	
Illiterate	2 ± 1.77
Elementary	48 ± 42.48
High school	34 ± 30.09
Superior	29 ± 25.66

**Table 2:** Baseline clinical and neurophysiological assessments according to polymorphism status.

	**OPRM1 A118G/rs1799971 (N=101)**			**OPRM1 C17T/ rs1799972 (N=101)**			**BDNF G196A/rs6265 (N=99)**		
**Variables**	**Carriers (N=22)**	**Non-carriers (N=79)**	**p value**	**Carriers (N=19)**	**Non-carriers (N=82)**	**p value**	**Carriers (N=27)**	**Non-carriers (N=72)**	**p value**
***Clinical***									
*OA Bilateral*	19 (100%)	73 (98.6%)	1.000	17 (100%)	75 (98.7%)	1.00	26 (96.1%)	66 (100%)	0.283
*Time of ongoing pain (months)*	60 (24 - 120).	60 (36-120)	0.778	60 (24 - 120)	60 (36 - 120)	0.655	60 (24 - 120)	60 (36 - 120)	0.640
*KL (average)*	2.45 (±1.17)	2.46 (±1.17)	0.968	2.56 (±1.12)	2.44 (±1.18)	0.673	1.9 (±1.13)	2.67 (±1.12)	**0.004**
*Pain (VAS)*	5.19 (± 2.53)	5.71 (±1.93)	0.309	5.68 (± 2.11)	5.58 (± 2.07)	0.857	5.16 (± 1.68)	5.83 (± 2.12)	0.141
*Pain (WOMAC)*	9.95 (±4.75)	11.22 (±3.69)	0.187	10.26 (± 4.51)	11.1 (± 3.83)	0.410	9.81 (±3.67)	11.56 (±3.80)	**0.044**
*Anxiety (HAD)*	5.90 (±4.72)	5.94 (±4.20)	0.977	5.56 (± 4.00)	6.01 (± 4.37)	0.685	4.88 (±4.10)	6.33 (±4.35)	0.143
*Depression (HDRS)*	8.14 (± 6.26)	9.61 (± 5.35)	0.283	8.33 (± 5.95)	9.52 (± 5.48)	0.416	9.15 (± 4.96)	9.44 (± 5.77)	0.820
*Catastrophizing (PCS)*	15.45 (±12.53)	13.90 (±10.64)	0.576	15.06 (±10.65)	14.02 (±11.14)	0.722	13.46 (±11.88)	14.62 (±10.76)	0.649
*MOCA*	22.35 (± 5.06)	20.55 (± 4.92)	0.150	20.72 (± 4.10)	20.96 (± 5.18)	0.854	21.27 (± 4.6)	20.73 (± 5.15)	0.641
***Neurophysiological***									
*MT*	51.89 (±8.41)	51.40 (±12.37)	0.863	55.53 (±10.4)	50.54 (±11.68)	0.09	54.52 (±12.19)	49.93 (±10.72)	0.073
*MEP*	1.99 (±2.17)	1.73 (±1.17)	0.478	1.74 (±1.08)	1.80 (±1.52)	0.882	1.58 (± 1.17)	1.85 (±1.53)	0.422
*SICI*	0.52 (±0.29)	0.45 (± 0.26)	0.331	0.47 (±0.23)	0.47 (±0.28)	0.909	0.43 (±0.22)	0.49 (±0.29)	0.349
*ICF*	1.80 (±0.59)	1.62 (±0.52)	0.165	1.70 (±0.70)	1.65 (±0.50)	0.720	1.63 (±0.63)	1.68 (±0.50)	0.723
*CSP*	82.2 (±31.42)	88.26 (±31.22)	0.426	89.21 (±31.58)	86.34 (±31.29)	0.721	82.90 (±24.31)	88.60 (±33.21)	0.420

MOCA: Montreal cognitive assessment; MT: motor threshold; MEP: motor-evoked potential; SICI: short-intracortical inhibition; ICF: intracortical facilitation; CSP: cortical silent period.

### Carriers versus non-carriers comparison at baseline

Table 2 summarizes the clinical and neurophysiological data between carriers and non-carriers of both *BDNF* (G196A) and *OPRM1* (A118G and C17T) polymorphisms. From baseline unadjusted comparisons, *BDNF* (G196A) polymorphism carriers had less pain than non-carriers (WOMAC pain scale), and less OA severity (Kellgren-Lawrence classification of OA). No other imbalances were found. After adjustment (for age, sex, disease severity, and depression), carriers showed less anxiety than non-carriers (β: -1.9, 95% CI -3.37 to -0.44; p = 0.011), and the significant association with pain and disease severity was lost. Alternatively, there were no baseline associations between the polymorphisms' status of the *OPRM1* gene (A118G/rs1799971 and C17T/ rs1799972) and pain-related variables or TMS outcomes, in the univariate or multivariate.

### Polymorphisms associations with outcome improvements after rehabilitation

#### OPRM1 polymorphisms

There were significant differences in the models analyzing improvement in clinical and neurophysiological outcomes after the rehabilitation treatment. From our adjusted models (for sex, age, OA severity, depression, and treatment duration), the A118G polymorphism carriers were less likely to reduce pain after treatment than non-carriers (OR: 0.15, 95% CI 0.04 to 0.60; p = 0.007), which represent 85% less odds of pain improvement for carriers. They also were less likely to increase SICI (e.g., increases % of inhibition) after treatment (OR = 0.30, 95% CI 0.10 to 0.92; p = 0.036). Similarly, T carriers of the C17T polymorphism of *OPRM1* were less likely to have larger CSP (e.g., increases of inhibition) after treatment (OR: 0.28, 95% CI 0.08 to 0.93; p = 0.038). The results did not change after further adjustment by anxiety levels. Interestingly, there were no significant results in the models for changes in cortical excitability (MT), corticospinal tract activity (MEP), and intracortical facilitation (ICF) (See Table 3).

**Table 3:** Odds ratios for the carriers of each polymorphism per outcome, for both adjusted and non-adjusted models.

Variables		OR (unadjusted)	95% CI	OR (adjusted) ƚ	95% CI	
**Pain improvement (VAS changes)**						
*OPRM1* (A118G)		0.35	[0.13 - 0.98]	0.15	[0.04 – 0.60] *	
*OPRM1* (C17T)		2.08	[0.54 - 8.01]	3.02	[0.68 – 13.46]	
*BDNF* (G196A)		0.41	[0.15 - 1.11]	0.28ǂ	[0.09 – 0.87] *	
**CSP improvement**						
*OPRM1* (A118G)		1.50	[0.56 - 4.05]	1.62	[0.58 – 4.53]	
*OPRM1* (C17T)		0.35	[0.11 - 1.10]	0.28	[0.08 – 0.93] *	
*BDNF* (G196A)		1.70	[0.65 - 4.41]	1.86	[0.66 – 5.26]	
**ICF improvement**						
*OPRM1* (A118G)		1.24	[0.46 - 3.32]	1.16	[0.39 – 3.45]	
*OPRM1* (C17T)		0.51	[0.17 - 1.55]	0.44	[0.13 –1.50]	
*BDNF* (G196A)		0.85	[0.33 - 2.18]	0.91	[0.32 – 2.60]	
**SICI improvement**						
*OPRM1* (A118G)		0.39	[0.14 - 1.10]	0.30	[0.10 – 0.92] *	
*OPRM1* (C17T)		1.12	[0.39 - 3.25]	0.90	[0.29 – 2.75]	
*BDNF* (G196A)		1.26	[0.49 - 3.24]	1.42	[0.51 – 3.96]	
**MT improvement**						
*OPRM1* (A118G)		0.65	[0.24 - 1.78]	0.61	[0.22 –1.73]	
*OPRM1* (C17T)		0.58	[0.19 - 1.73]	0.51	[0.16 – 1.60]	
*BDNF* (G196A)		1.30	[0.50 - 3.33]	1.75	[0.62 – 4.93]	
**MEP improvement**						
*OPRM1* (A118G)		0.97	[0.36 - 2.58]	0.87	[0.31 – 2.44]	
*OPRM1* (C17T)		0.94	[0.33 - 2.72]	0.87	[0.28 – 2.63]	
*BDNF* (G196A)		1.81	[0.70 - 4.71]	1.76	[0.63 – 4.94]	

OPRM1: opioid Receptor Mu 1 gene; BDNF: brain-derived neurotrophic factor.

ƚ Adjusted for sex, age, OA severity, depression, and treatment duration.

ǂ Adjusted for sex, age, OA severity, and depression.

⁎ Significant p values (p < 0.05).

#### BDNF polymorphism

We found that A carriers were less likely to improve their pain levels compared to non-carriers after the rehabilitation treatment (OR = 0.28, 95% CI 0.09 to 0.87 p = 0.028), which represent 72% less odds of pain improvement. No statistically significant associations with TMS metrics were found (Table 3).

### Polymorphisms moderation of pain and cortical inhibition responses

**Figure 1:**
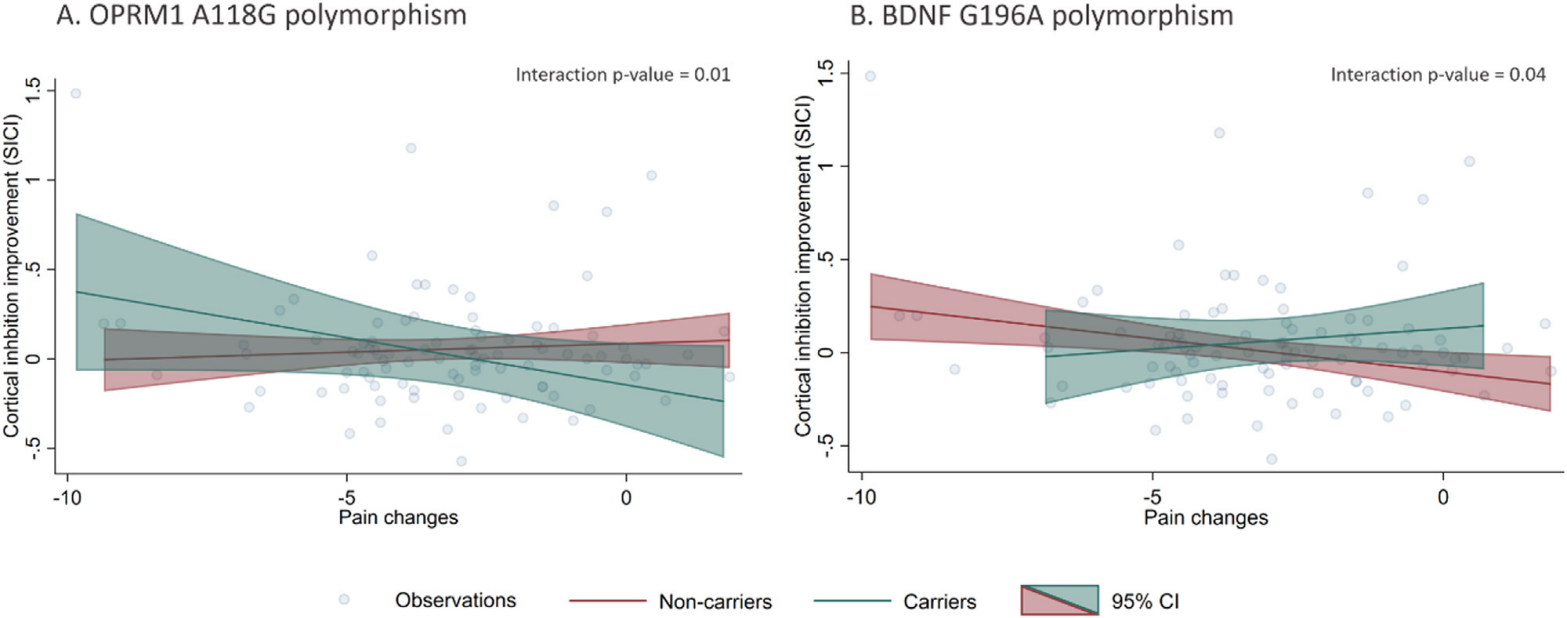
Moderation analysis. OPRM1 A118G and BDNF G196A polymorphisms moderates the association between pain and cortical inhibition responses after the rehabilitation treatment.

Our models did not show statistically significant correlations between VAS and cortical inhibitory metrics changes after treatment (pain changes vs. SICI changes: β = -0.01 p = 0.35; pain changes vs. CSP changes: β = 0.99 p = 0.30, respectively). However, when we added interaction terms for the polymorphisms and pain changes, we found statistically significant effect modifications (Table 4). The presence of OPRM1 A118G and BDNF G196A polymorphisms moderated the relationship between pain changes and SICI improvement. Individuals with the A118G variant allele had a negative association between SICI and pain differences (β: -0.05, p = 0.01), meaning carrier patients with higher cortical inhibition response had higher pain improvement (negative difference values post – pre). This relationship was not significant for the non-carrier subgroup (Fig. 1). Conversely, G196A non-carrier participants had a negative association between SICI and pain differences (β: -0.04, p = 0.01), meaning non-carrier with higher cortical inhibition response had higher pain improvement. This relationship was not significant for the carrier subgroup (Fig. 1). None of the other relationships between pain levels changes and the rest of inhibitory TMS measures were modified by the presence of the A118G, C17T *of OPRM1 gene* or G196A of *BDNF* gene polymorphisms.

**Table 4:** Effect modification by polymorphism status on the association of pain and cortical inhibition responses after rehabilitation.

TMS metrics	Simple slope in no-carriers#		Simple slope in carriers# #	Interaction beta coefficientƚ
**A118G *(OPRM1)***				
SICI	0.01 (p = 0.54)	-0.05 (p = 0.01)*		-0.06 (p = 0.010)*
CSP	0.59 (p = 0.63)	1.43 (p = 0.39)		0.84 (p = 0.68)
**C17T *(OPRM1)***				
SICI	-0.02 (p = 0.22)	0.01 (p = 0.82)		0.02 (p = 0.51)
CSP	0.97 (p = 0.35)	1.10 (p = 0.67)		0.13 (p = 0.96)
**G196A *(BDNF)***				
SICI	-0.04 (p = 0.01)*	0.02 (p = 0.38)		0.06 (p = 0.040)*
CSP	1.82 (p = 0.11)	-1.15 (p = 0.58)		-2.97 (p = 0.21)

# Relationship between pain changes and TMS metric changes after treatment in non-carriers.

# # Relationship between pain changes and TMS metric changes after treatment in carriers.

ƚ Difference of non-carriers and carriers slopes.

⁎ Significant (p<0.05).

## Discussion

### Main findings

We investigated the association of three single nucleotide polymorphisms (SNP) in chronic knee OA, two in the *OPRM1* gene (A118G and C17T) and one in the *BDNF* gene (G196A) with clinical characteristics at baseline, and treatment response (analgesic response and cortical inhibition improvement) after rehabilitation. We found that both *BDNF* and *OPRM1* polymorphisms carriers presented smaller analgesic response and cortical inhibition improvement after the rehabilitation program; but interestingly, the clinical and neurophysiological metrics among carriers and non-carriers were not different at baseline. Additionally, we found evidence of effect modification of both polymorphisms (A118G and G196A) on the association of pain changes and cortical inhibition improvement (SICI changes), showing that only among A118G carriers and G196A non-carriers the pain changes correlate with SICI changes – higher motor cortex inhibitory tonus, higher the analgesic response after rehabilitation.

### Polymorphisms and analgesic response after rehabilitation

#### Mu opioid receptor polymorphism

Polymorphisms in both genes are associated with augmented pain perception and the need of higher analgesic dose (Vossen et al., 2010), which is aligned to our results. We observed that patients with OA and *OPRM1* (A118G) polymorphism had the same baseline nociception as the others but developed a smaller analgesic response after rehabilitation compared to non-carriers. This is possibly associated with the loss of mu-opioid receptor (MOR) function found in G allele carriers. In vitro studies found that *OPRM1* (A118G) polymorphic patients presented less protein expression yields correlated with MOR function (Zhang et al., 2005). Moreover, a rat model G carrier study demonstrated that mutated rodents had less antinociceptive response to morphine and presented lower binding opioids in areas responsible for top-down pain inhibition, like the periaqueductal gray (PAG) (Wang et al., 2012).

Physical rehabilitation has been consolidated as a non-pharmacological strategy to decrease pain in OA patients (Bennell et al., 2012; Turner et al., 2020; Xie et al., 2021) – achieved by activating top-down pain inhibiting circuits (Lima et al., 2017). A central region of this neural pathway is the rostral ventral medulla (RVM) (Sluka et al., 2013), which is inhibited by the decrease in GABAergic activity in PAG (Bobeck et al., 2014). This suppression of GABAergic activity results from activating mu-opioid receptors in a presynaptic neuron in PAG (Bobeck et al., 2014). Since G carriers are associated with a decrease in MOR activity (REF), the lesser analgesic response after rehabilitation in mutated patients is possibly caused by the decreased MOR activity in the GABAergic neurons in the PAG of these patients. This would result in an unsuccessful suppression of the inhibitory projections that fire tonically from the PAG to the RVM,(Bobeck et al., 2014) leading this last area to continue to be inhibited even after the rehabilitation and, therefore, potentially deficient in decreasing pain.

#### BDNF polymorphism

We observed that patients with *BDNF* (G196A) polymorphism had a smaller analgesic response after the rehabilitation. BDNF is present throughout pain pathways (Lever et al., 2001; Merighi, 2018) and is related to pain modulation at supraspinal levels (Yue et al., 2017), including the activity of descending pain inhibition system mediated by endogenous opioids (Nijs et al., 2015). These descending pathways are stimulated by exercise and might be less activated in subjects with the polymorphism, as they are likely to have altered BDNF function (Hempstead, 2015; Notaras et al., 2015).

### Mu opioid receptor polymorphisms and cortical inhibition improvement

Our study is the first one to analyze intracortical inhibition in chronic knee OA pain patients and polymorphism in OPRM1 (A118 and C17T) and BDNF (G196A). We found that patients with A118G polymorphism had 76% less chance of increasing inhibition after treatment (0.3 OR), and that C17T participants also presented a lower chance of increasing measure cortical inhibition, in this case around 78% less (0.28 OR) then a nonpolymorphic participant. These findings were only related to SICI, but no other TMS metrics (MT, MEP, ICF) suggesting an inhibitory tonus specificity (GABAergic). Since these mutations are associated with decreased MOR activity and therefore lower GABA release at all times, this results could be interpreted as conflicting with the fact that mutated participants had no differences on cortical inhibition compared to non-carrier patients at baseline (Zhang et al., 2005). Although it is possible that compensatory mechanisms from indirect inhibitory pathways (non-dependent of OPRM1) could increase GABA concentration and therefore present similar cortical inhibition at baseline comparing carriers vs. non-carriers. However, after a rehabilitation treatment, the inhibitory changes are higher in the sensorimotor cortex from non-carriers individuals. We hypothesized that the extra sensorimotor engagement triggered by the rehabilitation program required larger inhibitory activation (higher recruitment of inhibitory network) which is disrupted in OPRM1 polymorphism carriers, thus these carriers have less intracortical inhibitory response (Dai et al., 2016; Ferguson & Gao, 2018). To understand the longitudinal differences across groups and to explore potential ceiling effects on inhibitory improvement, there is a need of studies with prolonged rehabilitation programs and longer follow-up.

Interestingly, we found different inhibitory markers associated with A118 and C17T polymorphisms, SICI and CSP, respectively. The C17T polymorphism of OPRM1 is considered the second most common coding region variant of OPRM1 gene, especially in Caucasians (Tan et al., 2003), but no previous studies have shown differential inhibitory profiles associated with A118 and C17T. We hypothesize that C17T could have associated with presynaptic GABAergic modulation which has been reported associated to CSP (McDonnell et al., 2006). However, further studies using molecular imaging are needed to test gabaergic network differences between different OPRM1 polymorphisms.

Additionally, we found evidence of effect measure modification of both polymorphisms (A118G and G196A) on the association of pain changes and cortical inhibition improvement (SICI changes), showing that only among A118G carriers and G196A non-carriers the pain changes correlates with SICI changes – higher motor cortex inhibitory tonus, higher the analgesic response after rehabilitation.

The effect modification by the presence of the OPMR1 (A118G) and *BDNF* (G196A) polymorphisms in the relationship between pain level improvements and SICI changes after treatment supports the search for biomarkers that are useful for directing pain treatments inducing neuroplastic changes. In carriers of OPMR1 (A118G) and non-carriers of the BDNF (G196A) polymorphism, changes in SICI were related to changes in pain, therefore, in those patients procedures aimed at modulating SICI could help alleviate pain, such as non-invasive brain stimulation (Cardenas-Rojas et al., 2020; Fregni et al., 2021; Pacheco-Barrios et al., 2020). Conversely, in A118G non-carriers and G196A carriers, increments in SICI were inversely related to improvements in pain: we can speculate that perhaps, in those individuals, other types of therapies would be better options.

### No clinical nor neurophysiological differences at baseline

At baseline, there was no significant difference for polymorphisms on *OPRM1*and *BNDF* with respect to pain intensity. A118G (rs1799971 and rs1799972) polymorphisms of *OPRM1* and BDNF (G196A) are just some of the genes related to pain perception, therefore, they cannot address completely the multidimensional mechanism of pain perception. Patients’ characteristics could explain variation in pain perception as age, sex, ethnicity, the genetic component, socioeconomic and psychological factors, among others (Foulkes & Wood, 2008). Besides, different single nucleotide polymorphisms (SNPs) have been studied for chronic pain and could act as potential unmeasured confounders. Also, it is important to notice that all included patients had chronic OA, thus, epigenetic compensatory mechanisms are likely in place balancing the clinical and neurophysiological characteristics between subgroups. Finally, our results show that these genes may play a larger role in explaining differences after dynamic neuroplasticity-induced changes in patients with chronic pain rather than baseline values. We hypothesize that if all patients in this cohort were engaged in constant regular rehabilitation programs before enrollment, then we would possibly see differences at the baseline.

### No effects on non-inhibitory TMS metrics

We did not detect differences for the polymorphisms regarding cortical excitability, measured by MT, corticospinal tract activity, measured by MEP, and intracortical facilitation (ICF) at baseline nor after the rehabilitation treatment. Motor threshold and MEP depends on the excitability of the neural network to propagate a signal (Hallett, 2007), it is known that voltage-gated sodium channels are the key on these connections (Ziemann, 2013). However, other polymorphisms related to sodium channels, such as SCN1A, have been associated with a change of excitability, hence the motor threshold (Menzler et al., 2014; Ogiwara et al., 2007). Neurotransmitter such as glutamate, GABA, dopamine, serotonin, acetylcholine and NE are suggested to impact MEP (Ziemann, 2013), while GABA and NE, intracortical facilitation. Our results did not provide evidence for the role of mu receptor polymorphisms in sodium and glutamate-related networks (like in MT, MEP, and ICF), however the association with polymorphisms in the pain pathway is still unclear (Berger et al., 2018; Moll et al., 2003; Ziemann et al., 1998).

### Limitations

We did not adjust for multiple comparisons due to the exploratory nature of this observational study and aiming to decrease type II error. However, we did limit the number of comparisons based on the biological plausibility of our hypotheses. In addition, the results were confirmed for different polymorphisms, strengthening the relevance of these findings. Still the hypotheses raised here must be confirmed in future studies.

## Conclusion

We found that in a knee OA sample with chronic pain, individuals with polymorphisms on both *BDNF* (G196A) and *OPRM1* (A118G) genes didn't differ at baseline but were less likely to improve pain after rehabilitation. Both variant alleles carriers of *OPRM1* polymorphisms were also less likely to improve cortical inhibition (SICI and CSP) in comparison to non-carriers. Moreover, while pain improvement and cortical inhibition improvement did not correlate in the total sample, the presence of *OPRM1* (A118G) and *BDNF* (G196A) polymorphisms moderated this relationship. Given that these genes are associated with neuroplasticity and our endogenous pain system, our results provide additional mechanistic evidence for the importance of neuroplasticity in triggering compensatory inhibitory mechanisms for chronic pain. Also, it underscored the promising role of combining genetic and neurophysiological markers to endotype the treatment response in chronic knee OA pain.

## Funding

This work was supported by a grant from FAPESP (SPEC project, fund number 2017/12943-8).

## Declaration of Interest Statement

No conflict of interest to declare. This study was developed in the absence of any commercial or financial relationships that could be construed as a potential conflict of interest.
